# The underlying mechanisms of arenaviral entry through matriglycan

**DOI:** 10.3389/fmolb.2024.1371551

**Published:** 2024-03-07

**Authors:** Michael Katz, Ron Diskin

**Affiliations:** Department of Chemical and Structural Biology, Weizmann Institute of Science, Rehovot, Israel

**Keywords:** lassa virus, arenaviruses, spike complex, LARGE1, receptor recognition, matriglycan

## Abstract

Matriglycan, a recently characterized linear polysaccharide, is composed of alternating xylose and glucuronic acid subunits bound to the ubiquitously expressed protein α-dystroglycan (α-DG). Pathogenic arenaviruses, like the Lassa virus (LASV), hijack this long linear polysaccharide to gain cellular entry. Until recently, it was unclear through what mechanisms LASV engages its matriglycan receptor to initiate infection. Additionally, how matriglycan is synthesized onto α-DG by the Golgi-resident glycosyltransferase LARGE1 remained enigmatic. Recent structural data for LARGE1 and for the LASV spike complex informs us about the synthesis of matriglycan as well as its usage as an entry receptor by arenaviruses. In this review, we discuss structural insights into the system of matriglycan generation and eventual recognition by pathogenic viruses. We also highlight the unique usage of matriglycan as a high-affinity host receptor compared with other polysaccharides that decorate cells.

## 1 Introduction

The cellular glycocalyx is a densely packed environment made of polysaccharides that extend out into the extracellular space. All human cells contain this external layer of sugars, which is critical for membrane remodeling, cellular morphology, and adherence to the extracellular matrix (ECM) (Shurer et al., 2019; Möckl, 2020). Long-chained polysaccharides such as heparan sulfate (HS), chondroitin sulfate (CS), and hyaluronan are known to decorate the cellular surface. These glycosaminoglycans (GAGs) interact with various binding partners from the ECM in addition to chemokines, growth factors, and proteases (Gandhi and Mancera, 2008). Another abundant polysaccharide that contributes to the eukaryotic glycocalyx is matriglycan. Although not containing an aminosugar and hence not considered a GAG, matriglycan is a GAG-like biopolymer that shares structural and chemical properties with other members of the glycocalyx (Sheikh et al., 2017; Hohenester, 2019). Matriglycan is uniquely present on the protein complex, dystroglycan, which is composed of an extracellular subunit, α-dystroglycan (α-DG) that interacts with ECM proteins, and a transmembrane subunit, β-dystroglycan, that binds to dystrophin via its C-terminal region (Jung et al., 1995; Endo, 2014). Dystroglycan plays a major role in the formation of the basement membrane and is essential in preserving healthy muscle function (Nickolls and Bönnemann, 2018). It connects ligands of the ECM with the actin cytoskeleton, thereby facilitating intracellular communication (Ervasti and Campbell, 1993).

In the early 1990s, the Campbell lab observed that the dystroglycan complex is highly glycosylated and interacts with laminin globular (LG) domains in the extracellular matrix (Ervasti and Campbell, 1991; Ibraghimov-Beskrovnaya et al., 1992; Ervasti and Campbell, 1993). It was later understood that α-DG displays matriglycan, a unique form of O-mannosylation (Chiba et al., 1997; Yoshida-Moriguchi and Campbell, 2015) containing the M3 core glycan (Ervasti and Campbell, 1993; Yoshida-Moriguchi et al., 2010; Yoshida-Moriguchi et al., 2013; Praissman and Wells, 2014). This extensive degree of α-DG’s glycosylation can account for over 50% of its total mass (Barresi and Campbell, 2006; Endo, 2014). Additionally, when these glycans on α-DG are not properly formed, debilitating phenotypes are known to occur, including types of congenital muscular dystrophies (CMDs) like Walker-Warburg syndrome (Longman et al., 2003; Godfrey et al., 2011). Impairments of α-DG glycosylation have also been linked to epithelium-derived cancers (de Bernabé et al., 2009; Quereda et al., 2022). Matriglycan is synthesized by the glycosyltransferase Like-acetylglucosaminyltransferase 1 (LARGE1) (Grewal et al., 2001; Barresi et al., 2004; Kanagawa et al., 2004) and is made of xylose (Xyl) and glucuronic acid (GlcA) in a long linear chain of repeating [-3GlcAβ1,3Xylα1-] heterodisaccharide subunits (Inamori and Hara, 2012; Goddeeris et al., 2013).

Matriglycan is covalently bound to two different motifs, Thr317/319 and Thr379/381, within the mucin-like domain of human α-DG (Yoshida-Moriguchi et al., 2010; Hara et al., 2011; Yang et al., 2023). Overall, more than 18 different enzymes are necessary for the proper formation of matriglycan (Beltrán et al., 2019; Hohenester, 2019; Kanagawa, 2021). The synthesis of full-length matriglycan is dependent upon LARGE1 recognition of the phosphorylation of the M3 glycan (Walimbe et al., 2020). LARGE1 must also be properly oriented through specific binding to the N-terminal domain of α-DG (Kanagawa et al., 2004; Fujimura et al., 2005; Okuma et al., 2023). Upon the sequential addition of β1,4-Xyl and β1,4-GlcA, by priming enzymes TMEM5 (Manya et al., 2016; Praissman et al., 2016) and B4GAT1 (Praissman et al., 2014; Willer et al., 2014), respectively, to the M3 core substrate, LARGE1 can initiate matriglycan synthesis (Sheikh et al., 2017; Endo, 2019).

Matriglycan plays a critical role in both cellular adhesion and differentiation (Moore and Winder, 2012; Goddeeris et al., 2013; Quereda et al., 2022), which is mediated by various laminin isoforms and other LG-domain-containing ECM proteins like agrin, perlecan, and neurexin (Hohenester, 2019). Interestingly, on top of its endogenous functions, matriglycan serves as the primary host receptor for highly pathogenic viruses. The Lassa virus (LASV), which causes the Lassa hemorrhagic fever (LHF) disease, as well as other viruses from the *Arenaviridae* family like the Lymphocytic choriomeningitis virus (LCMV), attach to this polysaccharide to initiate cell entry. Although α-DG itself was originally implicated to be the host receptor for the ‘Old World’ (OW) (*i.e.*, endemic to Africa) group of arenaviruses (Cao et al., 1998), the labs of Kunz (Kunz et al., 2005) and Oxenius and Brancaccio (Imperiali et al., 2005) found that it was the glycans on α-DG that facilitate this viral-host interaction. LASV is a pathogenic virus endemic to West Africa that infects hundreds of thousands of people annually (McCormick et al., 1987; McCormick and Fisher-Hoch, 2002; Hallam et al., 2018). The virus preferentially infects and replicates within macrophages, dendritic cells, and endothelial cells (Lukashevich et al., 1999; Baize et al., 2004; Schaeffer et al., 2018). Transmission typically occurs via the excrement of the *Mastomys natalensis* rat (Lecompte et al., 2006), but human-to-human transmission is also well documented (Fisher-Hoch et al., 1995; Lo Iacono et al., 2015; Akhmetzhanov et al., 2019). LASV infection can be fatal (McCormick et al., 1987; Yaro et al., 2021), and survivors are often left with permanent debilitating symptoms, such as deafness (Mateer et al., 2018) and vision impairment (Li et al., 2020). There is currently no FDA-approved vaccine against LASV nor any highly effective therapeutics (Eberhardt et al., 2019; Salam et al., 2022). It is, therefore, of major interest to elucidate the mechanisms used for arenaviral infection and to develop novel therapeutics against these pathogens.

How exactly α-DG-tropic arenaviruses recognize their host receptor has, until recently, remained a mystery. A wealth of knowledge has been generated over the past few decades, but challenges in isolating and visualizing an arenaviral spike have prevented a more holistic understanding. With the resolution revolution in cryo-EM (Kühlbrandt, 2014), we now have a clearer picture of the viral lifecycle of pathogenic viruses like LASV. This review will discuss recent developments, from a structural perspective, in determining how matriglycan is generated and later hijacked by arenaviruses for cellular entry.

## 2 Matriglycan: an infrequent glycocalyx entity with tissue-specific binding partners

Unlike most protein-based polysaccharides in the glycocalyx, matriglycan is almost exclusively found (besides several notable exceptions) (Inamori et al., 2016; Katz et al., 2022) covalently bound to a single protein, α-DG. The length of matriglycan is heterogeneous and tissue-specific (Goddeeris et al., 2013), but it is thought to contain at least 100 disaccharide repeats that extend off the surface of α-DG by more than ∼80 nm (Briggs et al., 2016; Hohenester, 2019). There seems to be preferential binding of certain laminin isoforms, like laminin 10/11, to shorter lengths of matriglycan (McDearmon et al., 2006). The ECM binding partners of matriglycan are effectively determined by their affinity to a specific polysaccharide length, where K_D_ values range between 1–100 nM (Goddeeris et al., 2013; Sciandra et al., 2013; Okuma et al., 2023). Thus, α-DG in particular tissues will have its preferred LG-domain containing ECM receptors.

A paralogue of LARGE1, called LARGE2 (from the *GYLTL1B* gene), also generates matriglycan yet has a narrower tissue distribution (Grewal et al., 2005; Ashikov et al., 2012; Inamori and Yoshida-Moriguchi, 2012). Collectively, these enzymes are expressed in a wide array of tissue types. As LARGE1 alone is thought to be sufficient for healthy tissue, LARGE2 is believed to often be dispensable, but it may function in a compensatory manner when LARGE1 is not expressed (Fujimura et al., 2005; Inamori et al., 2014). Although it has been implicated in serving a secondary role in maintaining α-DG glycosylation, recent studies have demonstrated abnormal activity of LARGE2 as a signature of colorectal cancer (Dietinger et al., 2020). Furthermore, aberrant expression of LARGE1/2, which has been detected in various types of cancers, may act to destabilize the binding between epithelial cells and the ECM (de Bernabé et al., 2009; Esser et al., 2013; Quereda et al., 2022). One notable difference between LARGE1 and its paralogue, LARGE2, is that the latter can generate matriglycan on other substrates. It was demonstrated that in α-DG^−/−^ embryonic stem cells, other heparan sulfate proteoglycans (HSPGs), such as biglycan and syndecan-2, could be modified by LARGE2 (Inamori et al., 2016). Therefore, LARGE2 likely has a broader specificity than LARGE1 and can act upon other proteoglycans rather than only on α-DG.

Matriglycan chain termination is carried out by the sulfotransferase HNK-1 through the addition of a sulfate group to the final GlcA residue (Sheikh et al., 2020). This terminal sulfate effectively eliminates the binding substrate of LARGE1/2 to prevent further matriglycan synthesis. Levels of HNK-1 expression are also highly heterogeneous and tissue-based. Thus, LARGE1, LARGE2, and HNK-1 likely function in parallel to generate specific matriglycan lengths that arise from the variable expression levels of these enzymes in each tissue. For instance, HNK-1 transcript abundance was found to be significantly higher in brain tissue, when compared to LARGE1/2, resulting in shorter matriglycan on α-DG (Goddeeris et al., 2013; Sheikh et al., 2020).

### 2.1 Mechanistic insights into LARGE1 synthesis of matriglycan

**FIGURE 1:**
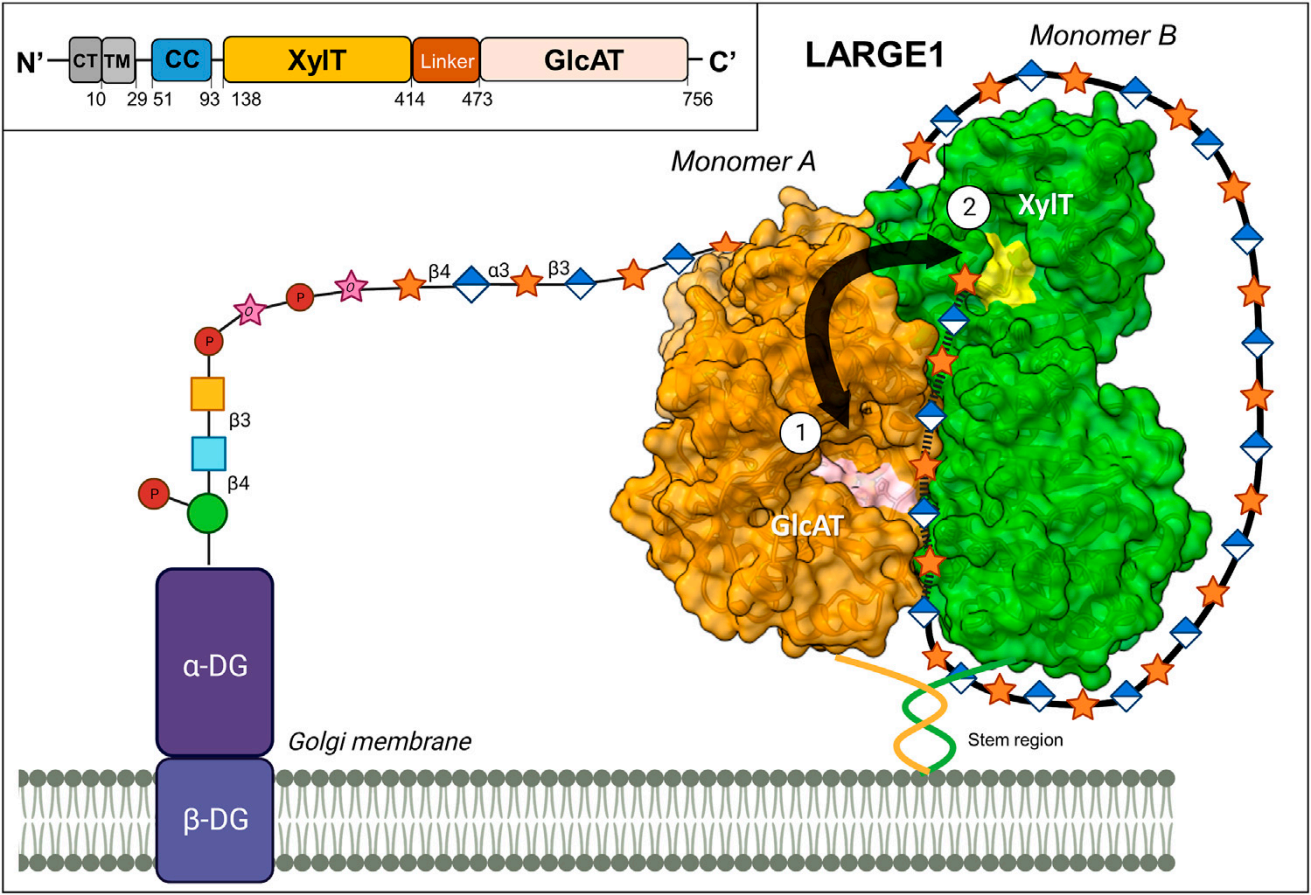
Proposed mechanism for matriglycan synthesis. LARGE1 is a homodimeric enzyme that synthesizes matriglycan in a processive manner on α-DG (PDB 7ZVJ). The growing polysaccharide chain alternates between the xylosyltransferase (XylT) domain on one monomer (shown in green), and the glucuronyltransferase (GlcAT) domain on the second monomer (shown in orange). The ‘DXD’ motifs, which comprise part of the catalytic sites, are colored in yellow and pink for the XylT and GlcAT domains, respectively. The matriglycan chain likely wraps around the perimeter of the enzyme, adhering to positively charged patches on the protein’s surface, aiding the enzyme’s processivity. Inset: A schematic diagram of the domain organization of LARGE1, CT: Cytoplasmic, TM: Transmembrane, CC: Coiled Coil. This image was partially created by BioRender.com.

LARGE1 is a bifunctional enzyme that is localized in the Golgi (Grewal et al., 2005; Percival and Froehner, 2007). This enzyme contains a cytoplasmic/transmembrane region, a coiled-coil (stem) domain, a xylosyltransferase (Xyl-T) domain, a linker region, and a glucuronyltransferase (GlcA-T) domain (Figure 1). The ectodomain structure of LARGE1 (residues 29–756) (Peyrard et al., 1999) was recently solved (Joseph et al., 2022; Katz and Diskin, 2022), providing insights into how matriglycan is synthesized. This enzyme exists as a homodimer, with two copies of each catalytic domain. Both catalytic domains consist of a canonical GT-A fold that contains an α/β/α sandwich, similar to a Rossman fold for nucleotide-binding proteins (Harrus et al., 2018; Taujale et al., 2020; Joseph et al., 2022; Katz and Diskin, 2022). Furthermore, the catalytic domains both contain a canonical ‘DXD’ motif, which forms part of each active site. This motif coordinates a divalent cation that interacts with the phosphate groups of the UDP-sugar donor (Breton et al., 2006; Taujale et al., 2020). The DXD motif of each domain straddles between the major and minor β-sheets where it engages with incoming monosaccharide-UDP substrates. The Xyl-T domain, a retaining transferase member of the GT 8 family (Drula et al., 2022), contains a DXD motif with a bound Mn^2+^ ion that facilitates the addition of a Xyl residue to the growing matriglycan chain (Katz and Diskin, 2022). The GlcA-T domain, an inverting transferase member of the GT 49 family (Drula et al., 2022), is predicted to coordinate a Mn^2+^ ion (or another divalent ion) in its DXD domain.

LARGE1 is organized in a parallel dimer. In this form, two LARGE1 monomers are associated such that each catalytic domain interacts with its cognate domain on the second monomer (Figure 1). Oligomerization in a parallel-dimeric form was found to be dependent upon the presence of the coiled-coil (CC) domain (Katz and Diskin, 2022). On each monomer, the two catalytic pockets face in opposite directions. Therefore, the closest distance between the two types of active sites is ∼40 Å, coming from catalytic domains on adjacent monomers. To access the two types of catalytic domains on the same monomer, the matriglycan chain would need to loop around to the opposite face of the enzyme after each subunit addition. Thus, the most efficient method for matriglycan generation is to use one domain contributed from each monomer in an alternating manner. Interestingly, several positively charged patches exist along the perimeter of the enzyme. These areas likely facilitate the growing (negatively charged) matriglycan to wrap around LARGE1 while having its non-reducing end stay near the two active sites (Katz and Diskin, 2022). Furthermore, LARGE1 synthesizes matriglycan processively, rather than distributively, through the use of these adjacent catalytic domains (Figure 1). The resulting matriglycan is generated in discrete lengths, which may be dictated by the inherent length of the enzyme-substrate complex (Joseph et al., 2022).

### 2.2 Architecture of the Lassa virus spike complex and mechanism for matriglycan recognition

**FIGURE 2:**
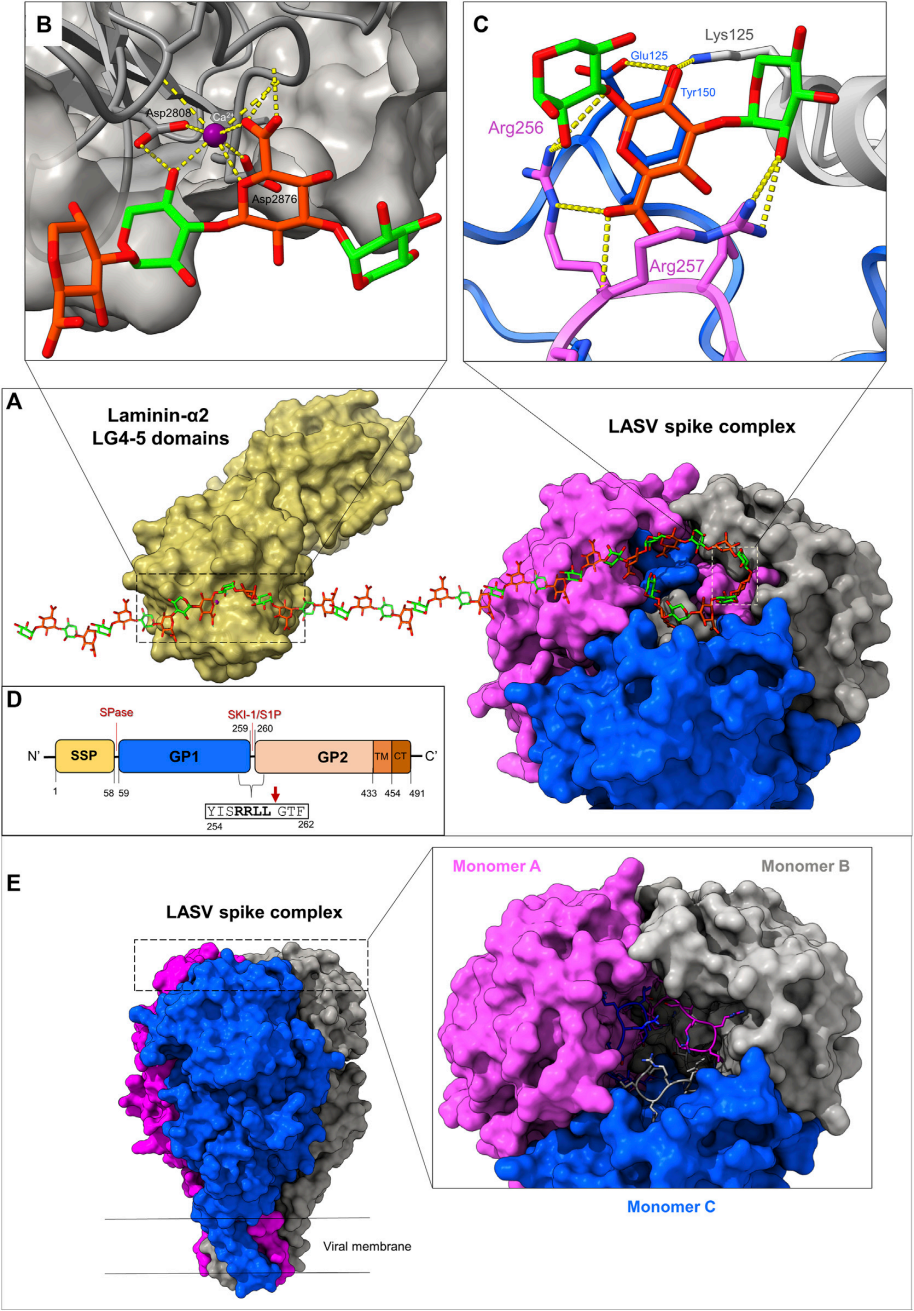
Mechanisms of matriglycan recognition by the LASV spike complex and LG4,5-domain-containing proteins. **(A)** Surface representations of the LG domains 4–5 of laminin α-2 chain, in complex with matriglycan (PDB 5IK5) alongside the Lassa spike complex, bound to matriglycan (PDB 7PVD). **(B)** The CA^2+^-dependent mechanism of the LG4-5 domain recognition of matriglycan, which chiefly relies on metal coordination. **(C)** The mechanism of LASV spike complex recognition of matriglycan, which depends on residues of a domain-swapped SKI-1/S1P site. This interaction is dominated by hydrogen bonds and electrostatic interactions. **(D)** A schematic diagram for the domain organization of the LASV spike complex. SSP: structured signal peptide, GP1: Glycoprotein-1 subunit, GP2: glycoprotein-2 subunit, TM: Transmembrane domain, CT: Cytoplasmic domain. The signal peptidase-1 (SPase) and SKI-1/S1P cleavage sites are indicated. The specific cleavage site by SKI-1/S1P is shown with a red arrow directly after the “RRLL” motif. Note that the use of a single matriglycan chain between the structures, along with only a partial description of key residues used for binding, was carried out for artistic purposes. **(E)** Stabilization of the LASV spike complex through domain swapping of the terminal GP1 regions (PDB: 7PUY). Monomers **(A–C)** of the trimeric spike complex are shown in pink, gray, and blue, respectively. The final ∼10 residues of GP1, which include the SKI-1/S1P recognition motif, are shown in stick representation. These segments are interlocked with a neighboring monomer through hydrophobic interactions, while exposing critical residues for matriglycan binding.

Arenaviruses are equipped with trimeric class-I spike complexes (Burns and Buchmeier, 1993; Burri and da Palma, 2012). Such spikes are translated as single polypeptide chains that trimerize and require maturation through proteolytic cleavage by cellular proteases to become active (Buchmeier and Parekh, 1987; Lenz et al., 2000; Schlie et al., 2010). The cleavage yields N-terminal receptor binding domains, termed glycoprotein-1 (GP1), C-terminal membrane fusion domains, termed glycoprotein-2 (GP2), and (unique to arenaviruses) a structured signal peptide (SSP) (Eichler et al., 2003; Burri and da Palma, 2012). For many years, it was unclear how arenaviruses like LASV utilize their spike complexes to bind the matriglycan receptor. The mechanism of matriglycan binding was recently revealed through the determination of the cryo-EM structure of a complete, trimeric LASV spike complex (Katz et al., 2022). The receptor binding site (RBS), where matriglycan binds, is quaternary and forms through the shared interactions between each of the spike’s three monomers at the apex of the spike (Figure 2A). At the heart of the binding site are the three C-terminal regions of the GP1 domains from each adjacent monomer, which undergo mutual domain-swapping with a neighboring subunit. This interlocked domain-swapping phenomenon provides structural stability and generates a surface-exposed cleft where matriglycan binds. Key residues that engage with matriglycan are contributed from the recognition sequence of the subtilisin kexin isozyme-1/site-1 (SKI-1/S1P) protease (*i.e.*, an “RRLL” sequence (Lenz et al., 2000)) that cleaves between the GP1 and GP2 subunits (Figure 2D). Although the general vicinity of the RBS was previously known through mutational analyses (Burri et al., 2013; Hastie et al., 2016; Acciani et al., 2017; Pennington and Lee, 2022), the function of the SKI-1/S1P recognition sequence for receptor binding (Katz et al., 2022) was unknown. This discovery helps to explain the exclusive use of the SKI-1/S1P protease by arenaviruses over more common cellular proteases, like furin, which is also considered to be a virulence-increasing factor in other viruses (Basak et al., 2001; Johnson et al., 2021).

Three equivalent matriglycan binding sites form on the apex of the spike. Since the RBS is generated through the interaction of the GP1 termini with neighboring monomers, it indicates that LASV cannot bind its host receptor unless the spike is fully matured (i.e., cleaved by SKI-1/S1P), rationalizing previous observations that soluble GP1 is alone unable to bind matriglycan (Jae et al., 2014; Cohen-Dvashi et al., 2015; Hastie et al., 2016; Acciani et al., 2017). Each binding site can accommodate two disaccharide repeats through the combined interactions of several key residues: Arg256, Arg257, and Leu258 (of the SKI-1/S1P site) from an “A”-monomer, and Tyr150 from a “B”-monomer (Figure 2C). In addition, GlcA subunits within matriglycan act as N-caps for short ⍺-helices on each GP1 that start with Leu120 and Ser121. Other important residues include Lys125 from an adjacent ‘C’-monomer that plays a critical role in positioning Tyr150 such that it can interact with a GlcA monosaccharide. Of these residues, Arg257 seems to be the most critical for matriglycan binding, as it interacts with both a GlcA and a Xyl residue, by making two hydrogen bonds through its guanidinium group and one via its main chain amine. Tyr150, which was recognized as an important residue for binding by genetic analyses (Hastie et al., 2016; Acciani et al., 2017), interacts directly with matriglycan and further stabilizes Arg257 through its main-chain carbonyl (Katz et al., 2022). Arg256 binds an entire disaccharide subunit and makes two hydrogen bonds with hydroxyl groups on matriglycan. The receptor binding site is preconfigured for binding through a series of other residues within GP1 that make secondary interactions. The bound matriglycan chain is approximately 40 Å in length and comprises 2,113 Å^2^ total buried surface area (903 Å^2^ within the RBS and 1,210 Å^2^ within matriglycan) (Katz et al., 2022). Interestingly, despite extensive N-linked glycosylation on the LASV spike (Watanabe et al., 2018), these glycans are not believed to play a role in matriglycan binding. Nonetheless, one particular N-linked glycan, Asn119, is highly conserved and may protect against immune detection of critical residues used for matriglycan binding (Sommerstein et al., 2015; Borenstein-Katz et al., 2019).

The molecular mechanism that LASV uses to bind matriglycan differs significantly from that of laminin G-like domains (Figure 2B). LG domains utilize a Ca^2+^ ion coordinated by two acidic side chains and main chain carbonyl groups to engage with matriglycan that completes an octahedral coordination geometry (Briggs et al., 2016). Additional residues of LG domains are involved in matriglycan binding, contributing to either direct or water-mediated polar interactions. The high affinity of LG domains to matriglycan may be due to the protein not requiring conformational changes for binding, reducing the binding’s entropic cost. Ligand binding, for both the LASV spike and LG domains, is facilitated by matriglycan being bound in a low-energy conformation with intramolecular hydrogen bonding within the polysaccharide chain. However, unlike LG domains, LASV does not engage in ion coordination and instead relies on a network of hydrogen bonding via positively charged residues in its SKI-1/S1P motif. The matriglycan binding interface of an LG domain is also much smaller, as only one Xyl-GlcA disaccharide repeat is bound, whereas LASV can fit at least five disaccharide subunits within the apex of its spike (Briggs et al., 2016; Katz et al., 2022).

### 2.3 The role of the SKI-1/S1P cleavage site in receptor recognition and the spike’s stability

Since the 1980s, it has been understood that arenaviral spikes require proteolytic maturation by the SKI-1/S1P protease for spike activation (Buchmeier et al., 1987) although SKI-1/S1P is not a common protease in the viral world (Lenz et al., 2000; Eichler et al., 2003). Besides arenaviruses, the use of the SKI-1/S1P protease has only been found in one other viral family, the *Nairoviridae* (which, like the Arenaviridae, is also in the *Bunyavirales* order) (Hulswit et al., 2021), where it activates the Gn envelope proteins of viruses like the Crimean-Congo hemorrhagic fever virus (CCHFV) (Vincent et al., 2003; Sanchez et al., 2006; Bost et al., 2023). In the absence of a functional SKI-1/S1P recognition motif, virions expressing the LCMV or the LASV spikes are no longer infectious (Lenz et al., 2001; Beyer et al., 2003). Even though arenaviruses can use other cellular receptors besides matriglycan, and thus do not necessarily need the SKI-1/S1P recognition motif for generating the receptor binding site, it seems that all human-infecting arenaviruses nevertheless contain a SKI-1/S1P proteolytic motif (Spiropoulou et al., 2002; Rojek and Lee, 2008; Pasquato et al., 2018). The Machupo virus (MACV), for instance, is a member of the New World (NW) arenaviruses (Charrel et al., 2002), which are primarily endemic to South America. MACV uses transferrin receptor 1 (TfR1) rather than matriglycan for cellular entry (Radoshitzky et al., 2007) in a mechanism that does not rely on the SKI-1/S1P recognition motif (Abraham et al., 2010; Diskin, 2023), but it preserves the SKI-1/S1P site for activation (Rojek and Lee, 2008). These observations suggest additional functions for the SKI-1/S1P recognition motif on top of forming the matriglycan binding site.

A possible hint for the additional functions of the SKI-1/S1P sites in arenaviral spike complexes comes from the extensive work leading up to obtaining structural information for the full-length LASV spike. Besides structural data for bits and pieces of these spikes (Bowden et al., 2009; Abraham et al., 2010; Igonet et al., 2011; Briknarová, 2012; Cohen-Dvashi et al., 2015; Hastie et al., 2016; Israeli et al., 2017; Shimon et al., 2017; Shulman et al., 2019; Zhang et al., 2019), low-resolution structural data for a complete spike first became available by EM subtomogram averaging from actual virions of the LASV, indicating its trimeric nature (Li et al., 2016). Later studies of the spike’s ectodomain required stapling antibodies to discern its architecture (Hastie et al., 2017) due to an apparent instability of these ectodomain constructs. However, an inherent stability of the spikes is a prerequisite for infectious particles that are transmitted through aerosol, as the spikes need to withstand the harsh external conditions required for transmission (Stephenson et al., 1984; Kenyon et al., 1992). Indeed, the WT full-length spike was found to be stable and hence suitable for structural studies (Katz et al., 2022). The cryo-EM structure of the LASV spike indicates that this stabilization originates from a two-sided interlocking of the protein complex (Katz et al., 2022). Inside the membrane, the GP2 helices are twisted around each other in a conformation that is locked by the SSP subunit, forming a stable six-helical bundle. At the apex of the spike, the SKI-1/S1P recognition motifs, contributed from each GP1 domain, interlocks the spike and stabilizes its trimeric form (Figure 2E). This additional identified role of the SKI-1/S1P recognition motif establishes a direct link between the spike’s stability and its ability to bind matriglycan and provides a possible explanation for its usage by non-matriglycan-tropic arenaviruses.

Interestingly, while all arenaviruses preserve the use of SKI-1/S1P for cleavage, sequence variations in the recognition motif do exist, and these variations may affect the maturation of newly formed spike complexes. SKI-1/S1P cleaves after hydrophobic residues in a general (K/R)-X-Xh-Xh motif (Seidah and Prat, 2007). The ‘P4’ site in arenaviruses has an invariant arginine residue, but the other positions do vary, mostly within the NW arenaviruses (Pasquato et al., 2011; Burri et al., 2013). The OW arenaviruses are relatively more conserved, composed of either “RRLL”, “RRLA”, or “RRLM” motifs (Burri et al., 2013). The “RRLL” motif is the consensus sequence of LASV strains (Lenz et al., 2000), and the mutation to “RRLA” in LASV prevents proteolytic processing (Burri and Pasqual, 2012). Although the evolutionary benefit of SKI-1/S1P sequence variation is unclear, a particular SKI-1/S1P sequence can dictate the intracellular compartments for post-translational processing (Burri et al., 2013). Noteworthy is that the LASV spike is cleaved in the ER/cis-Golgi, whereas the LCMV spike is cleaved in the late-Golgi (Beyer et al., 2003; Burri and Pasqual, 2012). When the SKI-1/S1P motif of LASV (‘RRLL’) is moved to LCMV’s spike, this mutant spike is then translocated to the ER/cis-Gogli as well (Burri and Pasqual, 2012; Oppliger and da Palma, 2016). Interestingly, the ‘RRLL’ motif of LASV mimics the SKI-1/S1P autoproteolysis site C and is the only known arenaviral motif that is identical to a cellular substrate (Pasquato et al., 2011; Burri and Pasqual, 2012). Most host substrates that are cleaved by SK1-1/S1P undergo processing in the medial Golgi, a distinct intracellular location relative to all known viral substrates. The alternative compartments for arenaviral processing may act to isolate viral spike maturation and thus prevent interruption of the processing of essential host proteins (Burri and Pasqual, 2012).

### 2.4 The lassa virus has high specificity for matriglycan

Many OW arenaviruses, and even several non-pathogenic NW arenaviruses, utilize matriglycan for cellular entry (Cao et al., 1998; Spiropoulou et al., 2002; Kunz et al., 2005; Rojek and Kunz, 2008). Virions of LASV and some LCMV strains engage matriglycan with high affinity, having reported half-maximal binding values of <10 nM (Cao et al., 1998; Kunz et al., 2004; Imperiali et al., 2005; Kunz et al., 2005; Kunz et al., 2005; Rojek and Sanchez, 2008). The binding strength between LASV and matriglycan likely stems from the extensive hydrogen bonding network that each spike forms with matriglycan and a capacity to bind several polysaccharide chains simultaneously over multiple spikes in the context of the complete virion. In addition, the C3-symmetry of each spike creates numerous degenerate binding states to the repeating linear matriglycan, which collectively decrease the entropic cost of binding (Lortat-Jacob et al., 2001; Erlendsson and Teilum, 2021; Katz et al., 2022). Kinetic data, alongside competition assays (Kunz et al., 2001; Rambukkana et al., 2003; Kunz et al., 2005), suggest that LASV (and some LCMV) virions have a matriglycan affinity on the same order of magnitude as many ECM proteins, inferring that arenaviruses can compete for free matriglycan in the extracellular environment. Matriglycan is relatively uncommon within the sea of glycans that surround the cellular membrane. (Pries et al., 2000; Reitsma et al., 2007; Gao and Lipowsky, 2010). Nonetheless, LASV has obtained specificity towards its polysaccharide receptor (Kunz et al., 2005; Rojek and Sanchez, 2008). Despite the similarities in structure, LASV and “high affinity” LCMV strains show minimal or no inhibition by soluble heparin (HP) and HS during cellular entry. LASV is seemingly unperturbed when infecting a cell line lacking HS- and chondroitin-containing proteoglycans in the glycocalyx (Rojek et al., 2007; Fedeli et al., 2018). Nonetheless, switching from matriglycan to other GAGs for cell entry is possible for some arenaviruses.

Unlike LASV, which exclusively utilizes matriglycan as a primary host receptor, some LCMV strains are able to use an alternative host receptor. This capability alters disease phenotypes and only requires minute changes in the spike’s sequence (Southern and Bishop, 1987; Suprunenko and Hofer, 2019). Specifically, while LCMV strains like cl-13 have high specificity for matriglycan (Kunz et al., 2004; Kunz et al., 2005), three mutations within the GP1 subunit of the LCMV spike complex are each independently sufficient for the virus to gain access to HS for cellular entry: Y155H (Hastie et al., 2016), S153F (Teng et al., 1996), L260F (Sevilla et al., 2000; Volland et al., 2021). Furthermore, these single-point mutations alter tissue tropism, with HS-tropic LCMV strains often being non-immunosuppressive and substantially less pathogenic (Salvato et al., 1991; Sevilla et al., 2000; Smelt et al., 2001). What is the evolutionary benefit of these LCMV strains that can enter cells via HSPGs, but do not generate disease? The gain of HSPG-mediated cell entry does allow for increased tropism for cells that have negligible matriglycan expression. Despite the ability of these LCMV strains to utilize another receptor, many of these variants have still maintained the capability to bind matriglycan, and some of them only utilize HS in the absence of α-DG (Kunz et al., 2004). Although LCMV receptor choice is not a direct predictor of disease severity, as there are exceptions like the highly pathogenic strain, WE2.2, it plays a critical role in determining the infected cell types and subsequent immune response (Teng et al., 1996; Kunz et al., 2004; Plume et al., 2019).

### 2.5 Viral entry through the use of GAGs and other glycans

High specificity binding to matriglycan by arenaviruses is not the only case for specific usage of linear glycans by viruses. While some viruses utilize GAGs as low-affinity attachment factors during cell entry (Xu and Esko, 2014), other viruses can generate high-affinity and tissue-specific interactions by taking advantage of the heterogeneous degree of N-sulfation, O-sulfation, and N-acetylation in the glycocalyx (Gallagher and Walker, 1985; Casu and Lindahl, 2001; Spillmann, 2001; Kobayashi et al., 2012). A subset of pathogenic viruses that engage HSPGs, like the Herpes Simplex Virus-1 (HSV-1) (Herold et al., 1995), rabies lyssavirus (Sasaki et al., 2018), and Dengue virus (DENV) (Chen et al., 1997), have a specific preference for the type/degree of HS sulfation (Xu and Esko, 2014). This specificity can result in high affinity, characterized by K_D_ values as high as 15 nM for DENV/HS (Chen et al., 1997), for example. Subsequently, viruses such as HSV-1 (O’Donnell et al., 2006; O’Donnell and Shukla, 2008; Madavaraju et al., 2020; Koganti et al., 2021), AAV-2 (Summerford and Samulski, 1998; Qiu et al., 2000), AcMNPV (Makkonen et al., 2013), and potentially DENV (Chen et al., 1997; Hilgard and Stockert, 2000; Dalrymple and Mackow, 2011; Cruz-Oliveira et al., 2015) may utilize GAGs alone for cell entry.

Noteworthy, besides long-linear polysaccharides, other types of cell-surface glycans could be used by viruses for cell entry. A known example is the influenza virus which uses α2-6-linked sialic acids for cellular entry via the hemagglutinin spikes on its viral surface. The affinity between one hemagglutinin spike to sialic acid is very low (0.5–5 mM) (Sauter et al., 1989; Gamblin and Skehel, 2010; Liu et al., 2022). However, influenza is equipped with 900–1,200 hemagglutinin trimers per virion, enabling numerous interactions with host sialic acids, resulting in a 10^4^–10^6^-fold increase in avidity (Kosik et al., 2018; Kosik and Yewdell, 2019). The sialic acid–binding adenovirus 37 (Ad37), for instance, is believed to increase its avidity 250-fold when it engages with a second sialic acid via a bivalent interaction (Nilsson et al., 2011). Thus, multivalent binding, which may act cooperatively, is used by many viruses to achieve high avidity interactions with their host receptors.

### 2.6 Cellular internalization via matriglycan

Recognition of the target cell via binding to the cell-surface receptor is a critical step for viral infection. However, merely attaching to the host cell is not sufficient, and viruses also need to penetrate cells either on the plasma membrane or alternatively inside some internal compartments following internalization. The usage of specific host receptors by viruses can alternate their cellular internalization/signaling pathways, as is the case for the human echovirus 1 with α2β1 integrin receptor (Jokinen et al., 2010; Boulant et al., 2015; Hussein et al., 2015; Salmela et al., 2017). Furthermore, other pathogens, like the poliovirus (Brandenburg et al., 2007; Coyne et al., 2007; Kalia and Jameel, 2011), modify their internalization strategy depending on the tissue being infected. In this regard, binding to matriglycan can facilitate two different pathways for internalization; LG domain-containing ECM proteins, like laminin-111, rely on a dynamin-dependent endocytic (pinocytotic) pathway for internalization (Leonoudakis et al., 2014) whereas α-DG tropic arenaviruses enter their host cell via the dynamin-independent process of macropinocytosis (Goncalves et al., 2013; Oppliger and Torriani, 2016). Numerous other viruses use macropinocytosis for cellular entry, such as HIV-1 (Liu et al., 2002), Vaccinia (Mercer and Helenius, 2008), and the Ebola virus (Mulherkar et al., 2011; Mercer and Helenius, 2012; Kuroda et al., 2020). Internalization via macropinocytosis is likely a form of apoptotic mimicry, in which cells detect phosphatidylserine (PS) on viral envelopes through PS-receptors, such as Axl, and Tyro3 (Shimojima et al., 2012; Shimojima and Kawaoka, 2012), that subsequently activate intracellular signaling cascades (Soares et al., 2008; Amara and Mercer, 2015). As macropinocytosis is used by the cell for the removal of apoptotic bodies, this pathway could be an effective way for viruses to evade immune detection, as it triggers intracellular pathways that modulate proinflammatory and anti-inflammatory cytokines (Mercer and Helenius, 2009; Amara and Mercer, 2015). For successful internalization, the virion needs to be in close proximity to the cell surface. How LASV or other matriglycan-tropic viruses ensure such proximity is not yet clear. An interesting possibility is that these viruses may slide on matriglycan toward the cellular membrane (*i.e.,* viral surfing) (Katz et al., 2022); whether or not this is the mechanism remains an open question.

### 2.7 Balancing cellular entry and viral egress

Given the abundance of a glycan receptor and the multiple spikes each virion has, the binding of the virion will have high avidity, potentially immobilizing viruses within the glycocalyx during cell exit. Some viruses, like HSV-1, overcome this issue by upregulating heparanase to the cellular surface to degrade heparan sulfate, and thus facilitating viral egress (Hadigal et al., 2015; Agelidis et al., 2017; Thakkar et al., 2017; Hadigal et al., 2020). Influenza, on the other hand, achieves viral egress by using neuraminidase to degrade sialic acids in the cellular periphery. Thus, the influenza virus requires a properly “tuned” hemagglutinin:neuraminidase ratio, ranging from 200:1 to 50:1 (dependent on the viral serotype and target tissue), in order to successfully escape the cell (Air and Laver, 1989; Harris et al., 2006; Xu et al., 2012; Gaymard et al., 2016). By contrast, LASV uses an entirely different mechanism for improving the likelihood of viral egress. Once new virions are formed within a host cell, they are equipped with a small fragment of matriglycan, which is bound within its spike complex (Katz et al., 2022). This “placeholder” would prevent LASV from binding with endogenous α-DG-bound matriglycan in the glycocalyx and re-infecting its host cell. Once the virion has successfully escaped, via budding (Murphy and Whitfield, 1975), to the extracellular environment (where free matriglycan is lacking), this receptor fragment is expected to dissociate so the virion can engage with another cell. An interesting open question is from where this piece of matriglycan-placeholder originates. This fragment may be synthesized by LARGE2 or the product of another yet-to-be-discovered cellular process.

## 3 Conclusion

The use of long-chained GAGs has historically been viewed as a general viral strategy to adhere to the host membrane through low affinity/non-specific interactions. Only recently has it become clear the complex role of these GAG interactions and the potential use of long-chained glycans as stand-alone host receptors. The Lassa virus engages in high-affinity interactions with its specific glycan receptor, matriglycan. Since the discovery that arenaviruses contain the rare SKI-1/S1P site within their viral spike complex, the preference for this particular proteolytic motif, as opposed to furin, has remained unclear. From the recent structure of the full-length native LASV spike complex, it is now understood that many OW arenaviruses contain this rare SKI-1/S1P motif because it serves the additional purpose of forming the matriglycan binding site. The very high degree of conservation of the SKI-1/S1P site among OW arenaviruses further validates the importance of this motif for viral entry (Burri et al., 2013; Katz et al., 2022). The additional role of the SKI-1/S1P sequence motif in stabilizing the trimeric form of the LASV spike complex may explain why all known human-infecting arenaviruses contain this site, while not all members of this viral family bind matriglycan. Understanding the structural basis for matriglycan recognition by LASV, and other arenaviruses, further paves the way to the design of new inhibitory molecules that will compete for the receptor binding site and thereby prevent viral attachment and entry. While we now have a better view of how pathogenic arenaviruses exploit matriglycan, many open questions remain regarding subsequent steps in the infection process.

## 4 Scope statement

A central attribute of the dystroglycan axis is matriglycan, the unique post-translational modification of α-dystroglycan. In this minireview, we discuss recent structural insights for the synthesis of matriglycan and the role of this polymeric sugar as a cell surface receptor for pathogenic viruses from the *Arenaviridae* family. Understanding how viruses, like the Lassa virus, recognize matriglycan provides invaluable data on the molecular mechanisms behind the infection process. The recently obtained structures of LARGE1, the enzyme responsible for matriglycan synthesis, and of the Lassa virus’s spike complex, bound to matriglycan, show how this polysaccharide is both generated and later hijacked by pathogens. Highlighting the unique role that the dystroglycan axis plays in infectious diseases is particularly relevant and would make an important contribution to this research topic.
